# Therapeutic potential of cladribine in combination with STAT3 inhibitor against multiple myeloma

**DOI:** 10.1186/1471-2407-11-255

**Published:** 2011-06-16

**Authors:** Jian Ma, Shuiliang Wang, Ming Zhao, Xin-Sheng Deng, Choon-Kee Lee, Xiao-Dan Yu, Bolin Liu

**Affiliations:** 1 International Medical Centre of PLA General Hospital, Beijing, PR China; 2 Department of Pathology, University of Colorado Anschutz Medical Campus School of Medicine, Aurora, CO, USA; 3 Department of Stress Medicine, Institute of Basic Medical Sciences, Beijing, PR China; 4 The Myeloma and Amyloidosis Program, Department of Medicine, University of Colorado Anschutz Medical Campus School of Medicine, Aurora, CO, USA

## Abstract

**Background:**

Cladribine or 2-chlorodeoxyadenosine (2-CDA) is a well-known purine nucleoside analog with particular activity against lymphoproliferative disorders, such as hairy cell leukemia (HCL). Its benefits in multiple myeloma (MM) remain unclear. Here we report the inhibitory effects of cladribine on MM cell lines (U266, RPMI8226, MM1.S), and its therapeutic potential in combination with a specific inhibitor of the signal transducer and activator of transcription 3 (STAT3).

**Methods:**

MTS-based proliferation assays were used to determine cell viability in response to cladribine. Cell cycle progression was examined by flow cytometry analysis. Cells undergoing apoptosis were evaluated with Annexin V staining and a specific ELISA to quantitatively measure cytoplasmic histone-associated DNA fragments. Western blot analyses were performed to determine the protein expression levels and activation.

**Results:**

Cladribine inhibited cell proliferation of MM cells in a dose-dependent manner, although the three MM cell lines exhibited a remarkably different responsiveness to cladribine. The IC50 of cladribine for U266, RPMI8226, or MM1.S cells was approximately 2.43, 0.75, or 0.18 μmol/L, respectively. Treatment with cladribine resulted in a significant G1 arrest in U266 and RPMI8226 cells, but only a minor increase in the G1 phase for MM1.S cells. Apoptosis assays with Annexin V-FITC/PI double staining indicated that cladribine induced apoptosis of U266 cells in a dose-dependent manner. Similar results were obtained with an apoptotic-ELISA showing that cladribine dramatically promoted MM1.S and RPMA8226 cells undergoing apoptosis. On the molecular level, cladribine induced PARP cleavage and activation of caspase-8 and caspase-3. Meanwhile, treatment with cladribine led to a remarkable reduction of the phosphorylated STAT3 (P-STAT3), but had little effect on STAT3 protein levels. The combinations of cladribine and a specific STAT3 inhibitor as compared to either agent alone significantly induced apoptosis in all three MM cell lines.

**Conclusions:**

Cladribine exhibited inhibitory effects on MM cells *in vitro*. MM1.S is the only cell line showing significant response to the clinically achievable concentrations of cladribine-induced apoptosis and inactivation of STAT3. Our data suggest that MM patients with the features of MM1.S cells may particularly benefit from cladribine monotherapy, whereas cladribine in combination with STAT3 inhibitor exerts a broader therapeutic potential against MM.

## Background

Multiple myeloma (MM) is a plasma cell malignancy characterized by specific genetic and epigenetic changes. Although many advances have been achieved in recent studies, MM remains an incurable disease and novel treatment strategies or agents are urgently needed [1,2]. A number of purine nucleoside analogs are rationally designed anticancer drugs that exert cytotoxicity via inhibition of DNA and RNA synthesis, and are currently used in the treatment of hematologic malignancies [3,4]. Cladribine (also known as 2-chlorodeoxyadenosine, 2-CDA) is an adenosine deaminase-resistant 2-deoxypurine nucleoside analog which requires phosphorylation by deoxycytidine kinase. Since this enzyme is mainly expressed in lymphocytes, cladribine is primarily active in lymphoid tissues [5]. Cladribine exerts remarkable activity in hairy cell leukemia (HCL), a chronic B-cell lymphoproliferative disorder, producing prolonged complete remissions in most patients [6,7]. Although cladribine is particularly cytotoxic to malignant B-cells and T-cells, and is widely used in HCL [8-10], it has not been approved to treat other lymphoid malignancies. Increasing evidences suggest that cladribine administered in combination with recently approved novel agents may be a valuable and safe treatment for patients with chronic lymphocutic leukemia (CLL) [11,12] and other lymphoid disorders, such as lymphoplasmacytic lymphoma, marginal zone lymphoma, and mantle cell lymphoma [13].

Although cladribine has been used for patients with low grade lymphoma and Waldenstrom's macroglobulinemia [14], it has only been studied in a limited manner in patients with MM, without much success [15]. Several studies have suggested that since "cladribine has a narrow spectrum of activity within the B-cell progeny" it may yet prove to be useful in subsets of patients with MM [16], because the self-renewing population of MM, arises at early B-cell precursors [17]. In vitro, the inhibitory effects of cladribine on MM cell lines are conflicting. While some studies observe completely negative results [18,19], others showing that cladribine has a marked heterogeneous effect on different MM cell lines [5] and clearly inhibits proliferation of RPMI8226 cells at high concentrations [20]. The precise molecular mechanisms by which MM cells show different responsiveness to cladribine remain unclear. It has been reported that cladribine induces accumulation of DNA strand breaks, and subsequently activates the tumor suppressor p53 in lymphocytes [21]. While mutation or deletion of *p53 *is rarely detected in untreated MM [22,23], it is not known whether *p53 *status in MM cell lines may influence their sensitivity to cladribine. Recent studies also suggest that frequent activation of STAT3 signaling provides survival advantage for MM cells [24-26], and STAT3 may serve as a novel target for the treatment of hematological tumors, including MM [27]. To date, there is no report indicating whether cladribine may modulate STAT3 activity in MM cells. Here, we studied cladribine's activity against different MM cell lines with either wild type (WT) or mutant *p53*, investigated its inhibitory effects on STAT3, and explored the therapeutic potential of cladribine in combination with a specific STAT3 inhibitor.

## Methods

### Reagents and antibodies

Cladribine or 2-chlorodeoxyadenosine (2-CDA) was purchased from Sigma-Aldrich Corp. (St. Louis, MO). STAT3 inhibitor VI (S3I-201) was obtained from EMD Chemicals, Inc. (Gibbstown, NJ). Antibodies for western blot analysis were from following sources: caspase-8 mouse mAb (1C12), caspase-9 polyclonal antibody, caspase-3 rabbit mAb (8G10), Poly (ADP-ribose) polymerase (PARP) rabbit mAb, phospho-STAT3 rabbit mAb and STAT3 (Cell Signaling Technology, Inc., Beverly, MA); b-actin mouse mAb (clone AC-75) (Sigma). All other reagents were purchased from Sigma unless otherwise specified.

### Cells and cell culture

Human MM cell line U266 was kindly provided by Dr. Lisheng Wang (Institute of Radiology, Academy of Military Medical Sciences, Beijing, China). Human MM cell line RPMI8226 was purchased from the American Type Culture Collection (ATCC, Manassas, VA). Human MM cell line MM1.S was kindly provided by Dr. Steven Rosen (Department of Medicine, Robert H. Lurie Comprehensive Cancer Center, Northwestern University, Chicago, IL). All cell lines were maintained in RPMI1640 cell culture medium supplemented with 10% fetal bovine serum (FBS) at a 37°C humidified atmosphere containing 95% air and 5% CO2 and were split twice a week.

### Cell proliferation assays

The CellTiter96™ AQ non-radioactive cell proliferation kit (Promega Corp., Madison, WI) was used to determine cell viability as we previously described [28]. In brief, cells were plated onto 96-well plates with either 0.1 ml complete medium (5% FBS) as control, or 0.1 ml of the same medium containing a series of doses of cladribine, and incubated for 72 hrs. After reading all wells at 490 nm with a micro-plate reader, the percentages of surviving cells from each group relative to controls, defined as 100% survival, were determined by reduction of MTS (3-(4,5-dimethylthiazol- 2-yl)-5-(3-carboxymethoxy phenyl)-2-(4-sulfophenyl)-2H-tetrazolium, inner salt).

### Flow cytometric analysis of cell cycle and apoptosis

Flow cytometric analyses were performed as described previously [28] to define the cell cycle distribution and apoptosis for treated and untreated cells. For cell cycle analysis, cells grown in 100-mm culture dishes were harvested and fixed with 70% ethanol. Cells were then stained for total DNA content with a solution containing 50 μg/ml propidium iodide and 100 μg/ml RNase A in PBS for 30 min at 37°C. Cell cycle distribution was analyzed with a FACScan flow cytometer (BD Biosciences, San Jose, CA). For apoptosis analysis, harvested cells were stained with Annexin V-FITC and propidium iodide according to the manufacturer's instruction and then subjected to the same analyzer.

### Quantification of apoptosis

An apoptosis ELISA kit (Roche Diagnostics) was used to quantitatively measure cytoplasmic histone-associated DNA fragments (mononucleosomes and oligonucleosomes) as previously reported [28].

### Western blot analysis

Protein expression levels were determined by western blot analysis as previously described [28]. Briefly, cells were lysed in a buffer containing 50 mM Tris, pH 7.4, 50 mM NaCl, 0.5% NP-40, 50 mM NaF, 1 mM Na3VO4, 1 mM phenylmethylsulfonyl fluoride, 25 μg/ml leupeptin, and 25 μg/ml aprotinin. The protein concentrations of the total cell lysates were determination by the Coomassie Plus protein assay reagent (Pierce Chemical Co., Rockford, IL). Equal amounts of cell lysates were boiled in Laemmli SDS-sample buffer, resolved by SDS-PAGE, transferred to nitrocellulose membrane (Bio-Rad Laboratories, Hercules, CA), and probed with specific antibodies as described in the figure legends. After the blots were incubated with horseradish peroxidase-labeled secondary antibody (Jackson ImmunoResearch Laboratories, Inc., West Grove, PA), the signals were detected using the enhanced chemiluminescence reagents (Amersham Life Science, Piscataway, NJ).

### Statistical analysis

Statistical analyses of the experimental data were performed using a two-sided Student's t test. Significance was set at a *P *< 0.05.

## Results

### Cladribine inhibits cell proliferation/survival of MM cells *in vitro*

**Figure 1:**
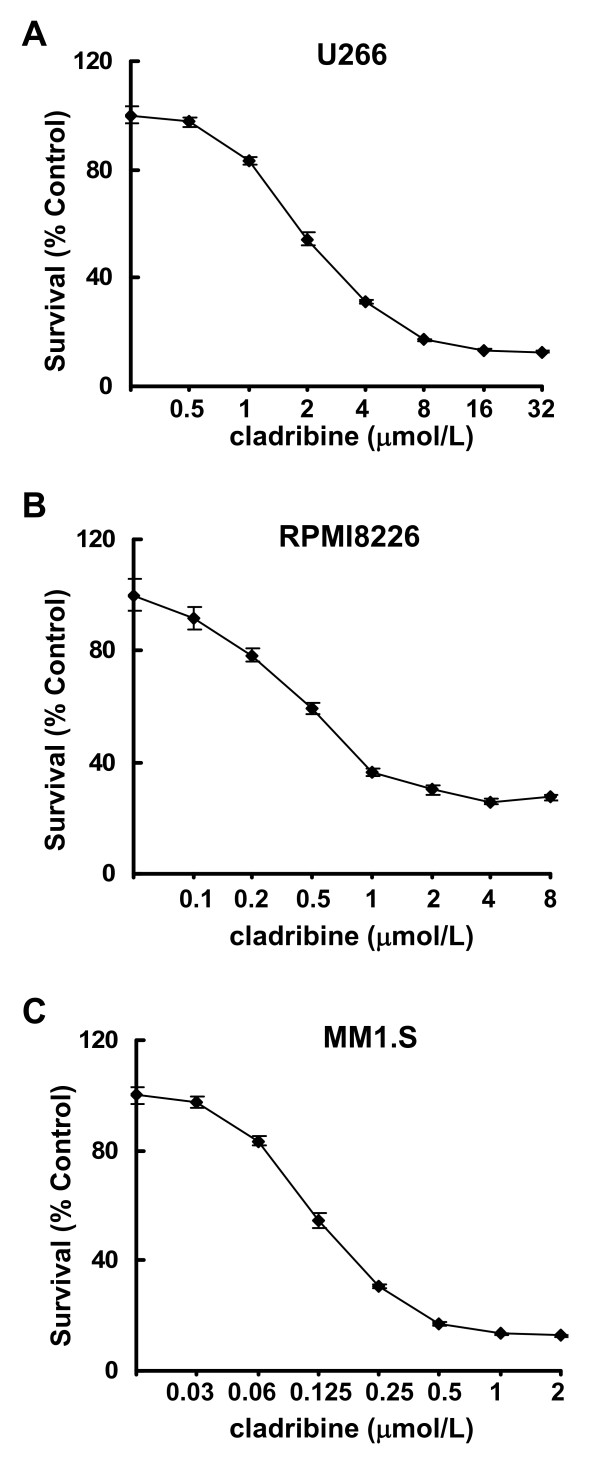
**Cladribine inhibits proliferation/survival of MM cells**. Human MM cells (1 × 10^4 ^cells/well) were plated onto 96-well plates with complete culture medium (RPMI1640, 10% FBS). After 24 hrs, the medium was replaced with fresh medium (0.5% FBS) or same medium containing indicated concentrations of cladribine for another 72 hrs. The percentages of surviving cells as compared to controls, defined as 100% survival, were determined by reduction of MTS. Data shows the representative of three independent experiments. *Bars*, SD. **A**, U266; **B**, RPMI8226; **C**, MM1.S

**Figure 2:**
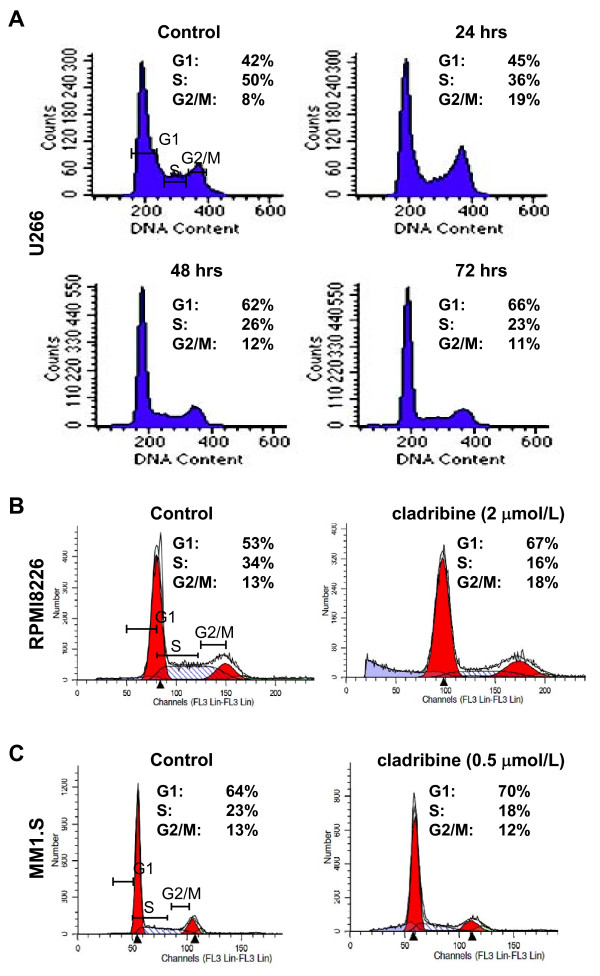
**Cladribine induces cell cycle G1 arrest in MM cells**. **A**, U266 cells were cultured with RPMI1640 (0.5% FBS) in the absence or presence of cladribine (2 μmol/L) for 24, 48, or 72 hrs. Cells were harvested and subjected to flow cytometry analysis of cell cycle distribution. Data shows the representative of three independent experiments. **B & C**, RPMI8226 (**B**) and MM1.S (**C**) cells cultured with RPMI1640 (0.5% FBS) in the absence or presence of cladribine for 24 hrs were harvested and subjected to flow cytometry analysis of cell cycle distribution. Data shows the representative of three independent experiments.

**Figure 3:**
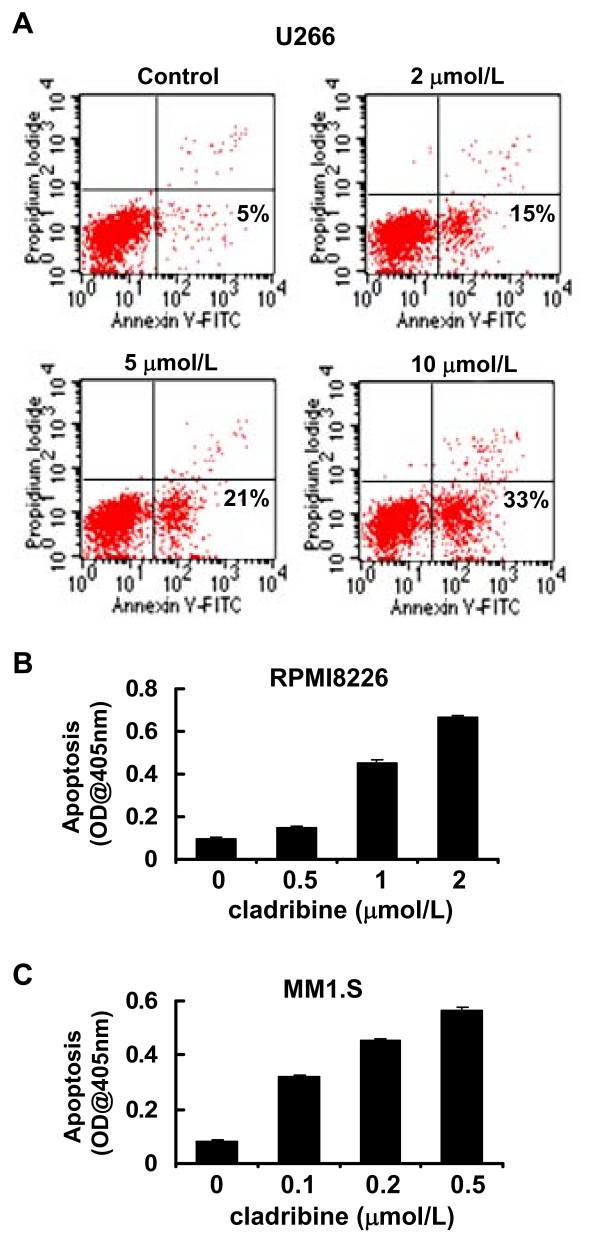
**Cladribine induces apoptosis in MM cells**. **A**, U266 cells were cultured with RPMI1640 (0.5% FBS) in the absence or presence of indicated concentrations of cladribine for 24 hrs. Cells were harvested and double-stained with Annexin V/PI, and then subjected to FACScan. The percentages of Annexin V-positive staining cells, indicative of apoptosis, were shown. **B & C**, RPMI8226 and MM1.S cells were cultured with RPMI1640 (0.5% FBS) in the absence or presence of indicated concentrations of cladribine for 24 hrs. Cells were collected and subjected to a specific apoptotic-ELISA. *Bars*, SD.

**Figure 4:**
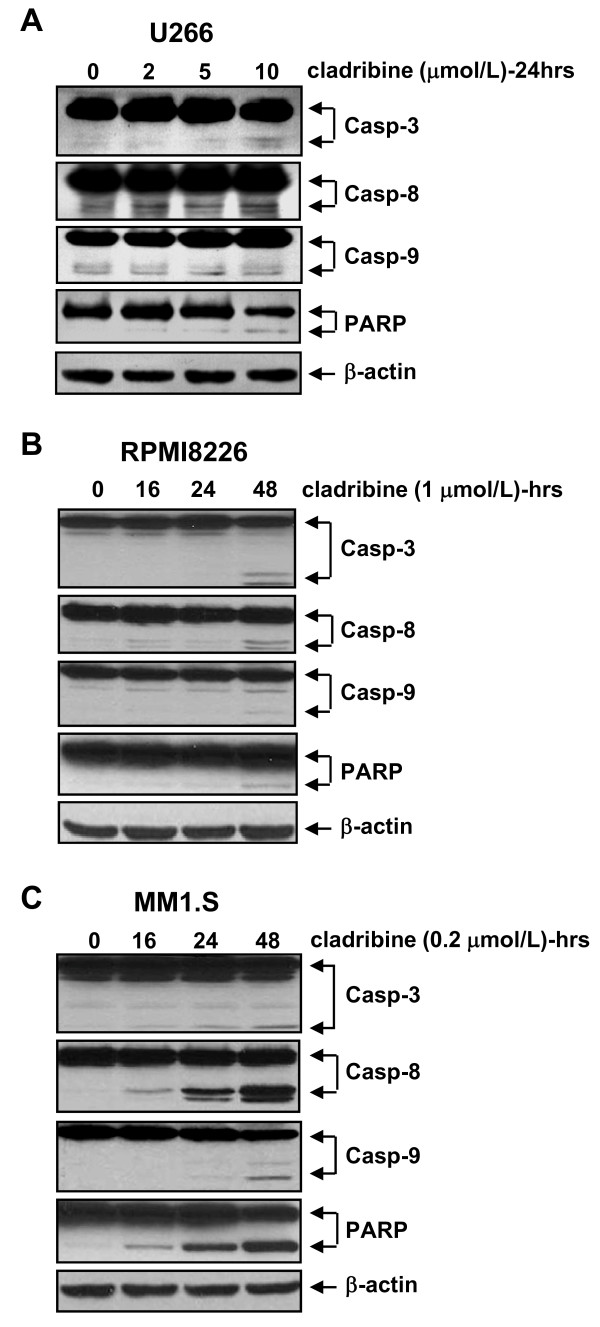
**Cladribine induces activation of caspase-3, -8, -9 and PARP cleavage in MM cells**. **A**, U266 cells were untreated or treated with indicated concentrations of cladribine for 24 hrs. **B & C**, RPMI8226 or MM1.S cells were untreated or treated with cladribine (1 μmol/L or 0.2 μmol/L, respectively) for 16, 24, or 48 hrs. Cells were collected and subjected to western blot analyses with specific antibodies directed against caspase-3 (Casp-3), caspase-8 (Casp-8), caspase-9 (Casp-9), PARP, or β-actin.

To explore whether cladribine might be a potential therapeutic agent against MM, we investigated its anti-proliferative/anti-survival effects on three MM cell lines: U266, RPMI8226 with mutant *p53*; and MM1.S which retains and expresses WT *p53 *[23]. Although the three MM cell lines exhibited different sensitivities, cladribine was able to inhibit proliferation/survival of all cells in a dose-dependent manner (Figure 1). While U266 was the least sensitive cell line, MM1.S was the most sensitive one to cladribine. The IC50s of cladribine for U266, RPMI8226, MM1.S cells were approximately 2.43, 0.75, and 0.18 μmol/L, respectively. To determine the molecular mechanisms by which cladribine inhibited proliferation/survival of MM cells, we first investigated the effects of cladribine on cell cycle progression. Both U266 and RPMI8226 cells with mutant *p53 *were treated with cladribine at the same concentration (2 μmol/L). U266 cells were collected at different time points (24, 48, or 72 hrs), and then analyzed with flow cytometry. Treatment with cladribine gradually increased the percentage of cells in the G1 phase of the cell cycle and reduced the percentage of cells in S phase (Figure 2A). Similar results were obtained in RPMI8226 cells with the treatment of cladribine for 24 hrs (Figure 2B). Cladribine appeared to increase G2-M phase in U266 cells upon 24 hr-treatment, it had no significant effect on G2-M phase either in U266 cells with 48 or 72 hr-treatment or in RPMI8226 cells (Figure 2A &2B). It remains unclear why cladribine affected G2-M phase in U266 cells only by 24 hr-treatment. MM1.S cells were treated with cladribine at a much lower concentration (0.5 μmol/L) for 24 hrs. Cladribine induced a minor increase in G1 phase, decreased the percentage cells in S phase, and had no effect on G2-M phase in MM1.S cells (Figure 2C). Although our cell proliferation assays indicated that the IC50 of cladribine was much lower for MM1.S cells than the IC50s for U266 and RPMI8226 cells (Figure 1), it appeared G1 arrest-induced by cladribine in MM1.S cells was not as profound as that we observed in the other two cell lines (Figure 2). It is likely that the potent anti-proliferative/anti-survival effects of cladribine on MM1.S cells may be mainly due to its strong capability to induce apoptosis as we discovered in the following studies (Figures 3 &4). Collectively, our data suggest that induction of cell cycle G1 arrest contributes to cladribine-mediated growth inhibition in MM cells.

### Cladribine induces apoptosis in MM cells

We next studied whether cladribine might also induce apoptosis in these MM cells, using two different methods. U266 cells were double-stained with Annexin V and propidium iodide, and then analyzed by a FACScan flow cytometer. These studies showed that cladribine induced apoptosis in U266 cells in a dose-dependent manner. The percentages of apoptotic cells evidenced by Annexin V-positive staining were 5%, 15%, 21%, and 33% when U266 cells were untreated or treated with 2, 5, 10 μmol/L of cladribine, respectively (Figure 3A). When an ELISA methodology was used to quantify apoptosis in RPMI8226 and MM1.S treated with cladribine, a dose-dependent increase in apoptosis was seen in both RPMI8226 and MM1.S cells (Figure 3B &3C). Consistent with the cell proliferation data (Figure 1), MM1.S was more sensitive to cladribine than RPMI8226 cells. To explore whether cladribine induced apoptosis through caspase-dependent mechanism, we carried out western blot assays to examine activation of caspases and PARP cleavage. In U266 cells, we were able to observe caspase-3 and caspase-8 activation and PARP cleavage only with cladribine at a higher concentration (10 μmol/L), however, it had no significant effect on caspase-9 activation (Figure 4A). Similar results were obtained in RPMI8226 cells treated with 1 μmol/L of cladribine for 48 hrs (Figure 4B). In contrast, treatment with cladribine at 0.2 μmol/L dramatically induced activation of caspase-3, -8, and -9 and PARP cleavage in a time-dependent manner in MM1.S (Figure 4C). Consistent with previous data derived from the apoptotic-ELISA (Figure 3B &3C), the lowest concentration of cladribine induced strongest activation of caspases and PARP cleavage in MM1.S cells (Figure 4). Taken together, our studies indicate that caspase-dependent apoptosis contributes to cladribine-mediated anti-proliferation/anti-survival effects on MM cells. Among the three MM cell lines tested, MM1.S is the most sensitive one to cladribine-induced apoptosis.

### Cladribine inactivates STAT3 signaling in MM cells

**Figure 5:**
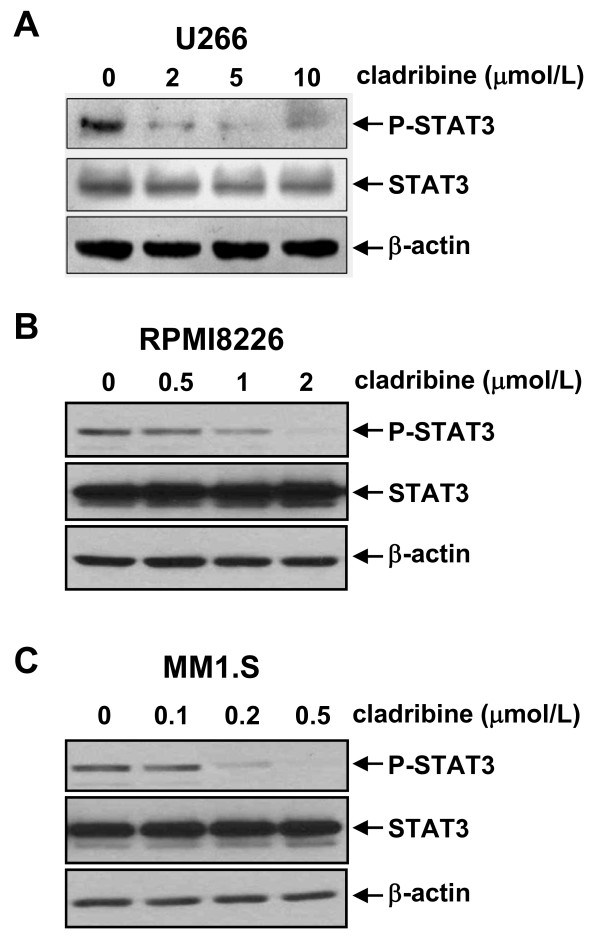
**Cladribine inactivates STAT3 in MM cells in a dose-dependent manner**. U266 (**A**), RPMI8226 (**B**), and MM1.S (**C**) cells untreated or treated with indicated concentrations of cladribine for 24 hrs collected and subjected to western blot analyses with specific antibodies directed against P-STAT3, STAT3, or β-actin.

It has been reported that constitutive activation of STAT3 is common in many human and murine cancer cells, and leads to cellular transformation [29,30]. Since aberrant activation of STAT3 plays a critical role in the development of human cancers, including MM [27], numerous studies have tried to identify novel anticancer strategies/agents targeting STAT3 [27,31]. To test whether cladribine's inhibitory activity against MM cells is due to STAT3 inactivation, we performed western blot analysis to determine the phosphorylation status of STAT3 in cladrabine-treated MM cells. In all three MM cell lines, cladribine significantly decreased the phospho-STAT3 (P-STAT3) levels in a dose-dependent manner, but had no effect on the total STAT3 protein levels (Figure 5). As with our cell proliferation (Figure 1) and apoptosis data (Figures 3 &4), treatment with low doses of cladribine (0.2 μmol/L) was as effective in reducing P-STAT3 in MM1.S cells (Figure 5C) as high doses were when applied to U266 (2 μmol/L) and RPMA8226 (1 μmol/L) cells (Figure 5A &5B). These data suggest that cladribine-induced growth inhibition and apoptosis in MM cells may be associated with its inactivation of STAT3.

### Combinations of cladribine and S3I-201, a specific STAT3 inhibitor, significantly promote MM cells undergoing apoptosis

**Figure 6:**
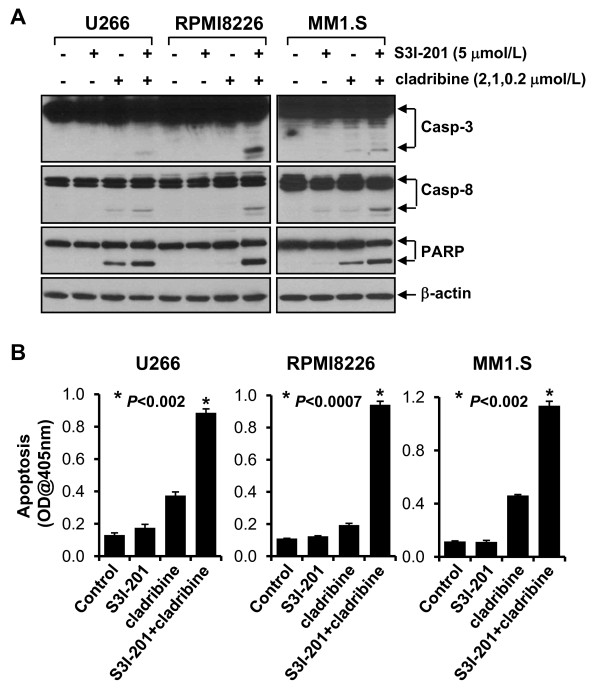
**The combinations of cladribine and S3I-201 significantly induce apoptosis in MM cells**. MM cells were untreated or treated with either S3I-201 (5 μmol/L), or cladribine (2 μmol/L for U266, 1 μmol/L for RPMI8226, 0.2 μmol/L for MM1.S) alone, or the combinations of S3I-201 and cladribine for 24 hrs. **A**, Cells were collected and subjected to western blot analyses with specific antibodies directed against caspase-3 (Casp-3), caspase-8 (Casp-8), PARP, or β-actin. **B**, Cells were collected and subjected to a specific apoptotic-ELISA. *Bars*, SD. *P *values vs single agent.

Since STAT3 activation is important in the development of human cancers, including MM [27], and cladribine was able to inhibit STAT3 in MM cells (Figure 5), we hypothesized that the combinations of cladribine and a specific STAT3 inhibitor might exhibit super activity in inducing apoptosis in MM cells. S3I-201, which selectively inhibits STAT3 DNA-binding activity [32], was chosen to test this hypothesis. It has been shown that treatment with 30 μmol/L of S3I-201 for 48 hrs induces significant apoptosis in human breast cancer cell line MDA-MB-435, which harbors constitutive active STAT3 [32]. S3I-201 with 5 μmol/L was used in the following assays, as this concentration alone did not induce apoptosis in all the three MM cell lines (Figure 6). In contrast, different concentrations of cladribine were used in the combinational studies: 2 μmol/L for U266 cells, 1 μmol/L for RPMI8226 cells, and 0.2 μmol/L for MM1.S cells, because treatment with cladribine at this concentration for 24 hrs did decrease P-STAT3 levels (Figure 5), but had no significant induction of caspase activation and PARP cleavage for each of the three MM cell lines (Figure 4). As expected, the combinations of cladribine and S3I-201 induced strong activation of caspase-3 and -8, and PARP cleavage in all three MM cell lines (Figure 6A). Furthermore, apoptotic-ELISA demonstrated that their combinations, as compared to either agent alone, significantly promoted MM cells undergoing apoptosis (Figure 6B, *P *< 0.002, *P *< 0.0007, *P *< 0.002 for U266, RPMI8226, MM1.S, respectively).

## Discussion

Although cladribine inhibited cell proliferation and induced apoptosis in all three MM cell lines tested, we used a wide range of concentrations of cladribine. Pharmacokinetic studies indicate that when given as a 2-hr bolus at a dose of 0.14 mg/kg, the mean peak plasma concentration of cladribine reaches 198 nmol/L and falls to 22.5 nmol/L within 6-hr [33,34]. The MM1.S cell line was the only one showing significant growth inhibition and apoptosis-induced by cladribine within this concentration range (Figures 1C, 3C, &4C). While our studies are consistent with a previous report indicating that cladribine has a heterogeneous effect on different MM cell lines [5], they suggest that cladribine may be useful to treat a subset of MM patients whose cells share similarities with MM1.S cells, which retain and express WT *p53 *[23]. In addition, like other clinically important nucleoside analogs, cladribine's effectiveness may be critically determined by the expression levels of deoxycytidine kinase (DCK), as this kinase is required to phosphorylate cladribine, and subsequently convert the inactive pro-drug into its active form [21]. We are currently testing whether cladribine may activate the tumor suppressor p53 in MM1.S cells, and whether or not this line expresses higher levels of DCK than U266 and RPMI8226 cells. Since cladribine at the clinically relevant concentrations dramatically reduced the levels of P-STAT3 in MM1.S cells (Figure 5C), this might serve as an *in vitro *screen for identifying potential cladribine candidates. These findings also suggest that cladribine-resistance may be attributed, in part, to a hyperactive STAT3 signaling pathway, which frequently occurs in MM [24-26]. In this report, we have focused our studies on modulation of STAT3 activity. Our data showed that the combinations of caldribine and S3I-201, a specific STAT3 inhibitor, indeed significantly induced apoptosis in all three MM cell lines (Figure 6).

Recent advances in identifying novel therapeutics against MM have provided new hope for this incurable disease. The inhibitors of histone deacetylase (HDAC) are promising agents for treatment of MM [35,36]. Our recent studies indicate that a class I HDAC inhibitor (HDACi), SNDX-275 exhibits strong anti-MM activities via enhanced DNA damage response and induction of apoptosis [28]. Although two HDACis, LBH589 and AR-42, have been shown to reduce STAT3 levels in human lung cancer cells and malignant mast cell disease, respectively [37,38], the effects of SNDX-275 on STAT3 activation and/or expression in MM cells remain unknow. It is not clear if SNDX-275 could reverse the cladribine resistant phenotype. It would be interesting and in clinical relevance to test the combinational activities of cladribine and SNDX-275 in MM.

It has been reported that the insulin-like growth factor-1 (IGF-1) and interleukin-6 (IL-6) are two major MM growth factors promoting cell proliferation and survival, and play a critical role in MM development [39,40]. Strategies targeting IGF-1 receptor (IGF-1R) - blocking antibodies and small molecule inhibitors - show very encouraging preclinical results against MM cells [41], and both strategies are now in clinical trials [42]. IGF-1 and IL-6 binds their specific receptors and subsequently result in activation of several signal transduction pathways [35], including the JAK/STAT3, PI-3K/Akt, Ras/MAPK, NF-κB and β-catennin pathway. The PI-3K/Akt signaling is a well-known cell survival pathway, and its activation often leads to resistance to therapeutic agents in cancer treatment [43,44]. Currently, it is unclear whether the autocrine or paracrine IGF-1/IGF-1R loop in MM and through which downstream signaling pathways may also contribute to cladribine-resistance as we observed in U266 and RPMI8226 cells.

## Conclusions

Cladribine-induced growth inhibition and apoptosis in MM cells correlated with its ability to inactivate STAT3. Cladribine in combination with S3I-201, a specific STAT3 inhibitor, resulted in significant apoptosis in all three MM cell lines as compared to either agent alone. Although cladribine as a single agent seems active in MM cells with WT *p53*, our studies suggest that the combinational regimens consisting of cladribine and STAT3 inhibitors may be more promising for MM patients.

## List of abbreviations

MM: multiple myeloma; HCL: hairy cell leukemia; CLL: chronic lymphocutic leukemia; 2-CDA: 2-chlorodeoxyadenosine; STAT3: signal transducer and activator of transcription 3; JAK: Janus kinase; IL-6: interleukin-6; DCK: deoxycytidine kinase; IGF-1: insulin-like growth factor-1; IGF-1R: IGF-1 receptor; PI-3K: phosphoinositide 3-kinase; MAPK: mitogen-activated protein kinase; NF-κB: nuclear factor-kappa B; HDAC: histone deacetylase; HDACi: inhibitor of HDAC; PARP: poly(ADP-ribose) polymerase; ELISA: enzyme-linked immunosorbent assay; PAGE: polyacrylamide gel electrophoresis

## Competing interests

The authors declare that they have no competing interests.

## Authors' contributions

The authors' contributions to this research work are reflected in the order shown, with the exception of BL who supervised the research and finalized the report. JM, SW, and MZ carried out all of the experiments. JM and BL drafted the manuscript. XSD, CKL, XDY, and BL conceived of the study, and participated in its design and coordination. All authors read and approved the final manuscript.

## Pre-publication history

The pre-publication history for this paper can be accessed here:

http://www.biomedcentral.com/1471-2407/11/255/prepub

## References

[B1] Kyle RA Rajkumar SV Multiple myeloma N Engl J Med 2004 351 1860 1873 10.1056/NEJMra041875 15509819

[B2] Naumann F Weingart O Kruse E Schulz H Bohlius J Hulsewede H Engert A Fifth biannual report of the cochrane haematologic malignancies group--focus on multiple myeloma J Natl Cancer Inst 2006 98 E2 E 10.1093/jnci/djj328 16912255

[B3] Robak T Korycka A Kasznicki M Wrzesien-Kus A Smolewski P Purine nucleoside analogues for the treatment of hematological malignancies: pharmacology and clinical applications Curr Cancer Drug Targets 2005 5 6 421 444 10.2174/1568009054863618 16178817

[B4] Robak T Lech-Maranda E Korycka A Robak E Purine nucleoside analogs as immunosuppressive and antineoplastic agents: mechanism of action and clinical activity Curr Med Chem 2006 13 3165 3189 10.2174/092986706778742918 17168705

[B5] Hjertner O Borset M Waage A Comparison of the effects of 2-chlorodeoxyadenosine and melphalan on myeloma cell lines Leuk Res 1996 20 155 160 10.1016/0145-2126(95)00128-X 8628014

[B6] Gidron A Tallman MS 2-CdA in the treatment of hairy cell leukemia: a review of long-term follow-up Leuk Lymphoma 2006 47 11 2301 2307 10.1080/10428190600822052 17107901

[B7] Huynh E Sigal D Saven A Cladribine in the treatment of hairy cell leukemia: initial and subsequent results Leuk Lymphoma 2009 50 Suppl 1 12 17 19814692 10.3109/10428190903142083

[B8] Chadha P Rademaker AW Mendiratta P Kim B Evanchuk DM Hakimian D Peterson LC Tallman MS Treatment of hairy cell leukemia with 2-chlorodeoxyadenosine (2-CdA): long-term follow-up of the Northwestern University experience Blood 2005 106 241 246 10.1182/blood-2005-01-0173 15761021

[B9] Cheson BD Sorensen JM Vena DA Montello MJ Barrett JA Damasio E Tallman M Annino L Connors J Coiffier B Lauria F Treatment of hairy cell leukemia with 2-chlorodeoxyadenosine via the Group C protocol mechanism of the National Cancer Institute: a report of 979 patients J Clin Oncol 1998 16 3007 3015 9738569 10.1200/JCO.1998.16.9.3007

[B10] Jehn U Bartl R Dietzfelbinger H Haferlach T Heinemann V An update: 12-year follow-up of patients with hairy cell leukemia following treatment with 2-chlorodeoxyadenosine Leukemia 2004 18 1476 1481 10.1038/sj.leu.2403418 15229616

[B11] Bertazzoni P Rabascio C Gigli F Calabrese L Radice D Calleri A Gregato G Negri M Liptrott SJ Bassi S Nassi L Sammassimo S Laszlo D Preda L Pruneri G Orlando L Martinelli G Rituximab and subcutaneous cladribine in chronic lymphocytic leukemia for newly diagnosed and relapsed patients Leuk Lymphoma 2010 51 1485 1493 10.3109/10428194.2010.495799 20578816

[B12] Leupin N Schuller JC Solenthaler M Heim D Rovo A Beretta K Gregor M Bargetzi MJ Brauchli P Himmelmann A Hanselmann S Zenhäusern R Efficacy of rituximab and cladribine in patients with chronic lymphocytic leukemia and feasibility of stem cell mobilization: a prospective multicenter phase II trial (protocol SAKK 34/02) Leuk Lymphoma 2010 51 613 619 10.3109/10428191003624231 20218808

[B13] Sigal DS Miller HJ Schram ED Saven A Beyond hairy cell: the activity of cladribine in other hematologic malignancies Blood 2010 116 2884 2896 10.1182/blood-2010-02-246140 20634380

[B14] Dimopoulos MA Kantarjian H Estey E O'Brien S Delasalle K Keating MJ Freireich EJ Alexanian R Treatment of Waldenstrom macroglobulinemia with 2-chlorodeoxyadenosine Ann Intern Med 1993 118 195 198 8093333 10.7326/0003-4819-118-3-199302010-00007

[B15] Dimopoulos MA Kantarjian HM Estey EH Alexanian R 2-Chlorodeoxyadenosine in the treatment of multiple myeloma Blood 1992 80 1626 1355673

[B16] Niesvizky R Siegel D Michaeli J 2-Chlorodeoxyadenosine for multiple myeloma Blood 1993 81 868 869 7679003

[B17] Niesvizky R Siegel D Michaeli J Biology and treatment of multiple myeloma Blood Rev 1993 7 24 33 10.1016/0268-960X(93)90021-U 8467229

[B18] Krett NL Ayres M Nabhan C Ma C Nowak B Nawrocki S Rosen ST Gandhi V In vitro assessment of nucleoside analogs in multiple myeloma Cancer Chemother Pharmacol 2004 54 113 121 15133625 10.1007/s00280-004-0777-2

[B19] Nagourney RA Evans SS Messenger JC Su YZ Weisenthal LM 2 chlorodeoxyadenosine activity and cross resistance patterns in primary cultures of human hematologic neoplasms Br J Cancer 1993 67 10 14 10.1038/bjc.1993.3 8094002 PMC1968239

[B20] Bagley RG Roth S Kurtzberg LS Rouleau C Yao M Crawford J Krumbholz R Lovett D Schmid S Teicher BA Bone marrow CFU-GM and human tumor xenograft efficacy of three antitumor nucleoside analogs Int J Oncol 2009 34 1329 1340 19360345

[B21] Johnston JB Mechanism of Action of Pentostatin and Cladribine in Hairy Cell Leukemia Leuk Lymphoma 2011 10.3109/10428194.2011.570394 21463108

[B22] Chesi M Bergsagel PL Epigenetics and microRNAs combine to modulate the MDM2/p53 axis in myeloma Cancer Cell 2010 18 299 300 10.1016/j.ccr.2010.10.004 20951939

[B23] Pichiorri F Suh SS Rocci A De Luca L Taccioli C Santhanam R Zhou W Benson DM Jr Hofmainster C Alder H Downregulation of p53-inducible microRNAs 192, 194, and 215 impairs the p53/MDM2 autoregulatory loop in multiple myeloma development Cancer Cell 2010 18 367 381 10.1016/j.ccr.2010.09.005 20951946 PMC3561766

[B24] Burger R Bakker F Guenther A Baum W Schmidt-Arras D Hideshima T Tai YT Shringarpure R Catley L Senaldi G Gramatzki M Anderson KC Functional significance of novel neurotrophin-1/B cell-stimulating factor-3 (cardiotrophin-like cytokine) for human myeloma cell growth and survival Br J Haematol 2003 123 869 878 10.1046/j.1365-2141.2003.04686.x 14632778

[B25] Catlett-Falcone R Landowski TH Oshiro MM Turkson J Levitzki A Savino R Ciliberto G Moscinski L Fernandez-Luna JL Nunez G Dalton WS Jove R Constitutive activation of Stat3 signaling confers resistance to apoptosis in human U266 myeloma cells Immunity 1999 10 105 115 10.1016/S1074-7613(00)80011-4 10023775

[B26] Hodge DR Xiao W Wang LH Li D Farrar WL Activating mutations in STAT3 and STAT5 differentially affect cellular proliferation and apoptotic resistance in multiple myeloma cells Cancer Biol Ther 2004 3 188 194 14726660 10.4161/cbt.3.2.621

[B27] Al Zaid Siddiquee K Turkson J STAT3 as a target for inducing apoptosis in solid and hematological tumors Cell Res 2008 18 254 267 10.1038/cr.2008.18 18227858 PMC2610254

[B28] Lee CK Wang S Huang X Ryder J Liu B HDAC inhibition synergistically enhances alkylator-induced DNA damage responses and apoptosis in multiple myeloma cells Cancer Lett 2010 296 233 240 10.1016/j.canlet.2010.04.014 20447761 PMC2990271

[B29] Bromberg JF Wrzeszczynska MH Devgan G Zhao Y Pestell RG Albanese C Darnell JE Jr Stat3 as an oncogene Cell 1999 98 295 303 10.1016/S0092-8674(00)81959-5 10458605

[B30] Yu H Jove R The STATs of cancer--new molecular targets come of age Nat Rev Cancer 2004 4 97 105 10.1038/nrc1275 14964307

[B31] Yue P Turkson J Targeting STAT3 in cancer: how successful are we? Expert Opin Investig Drugs 2009 18 45 56 10.1517/13543780802565791 19053881 PMC2610472

[B32] Siddiquee K Zhang S Guida WC Blaskovich MA Greedy B Lawrence HR Yip ML Jove R McLaughlin MM Lawrence NJ Selective chemical probe inhibitor of Stat3, identified through structure-based virtual screening, induces antitumor activity Proc Natl Acad Sci USA 2007 104 7391 7396 10.1073/pnas.0609757104 17463090 PMC1863497

[B33] Liliemark J Juliusson G On the pharmacokinetics of 2-chloro-2'-deoxyadenosine in humans Cancer Res 1991 51 5570 5572 1680554

[B34] Sonderegger T Betticher DC Cerny T Lauterburg BH Pharmacokinetics of 2-chloro-2'-deoxyadenosine administered subcutaneously or by continuous intravenous infusion Cancer Chemother Pharmacol 2000 46 40 42 10.1007/s002800000129 10912576

[B35] Hideshima T Mitsiades C Tonon G Richardson PG Anderson KC Understanding multiple myeloma pathogenesis in the bone marrow to identify new therapeutic targets Nat Rev Cancer 2007 7 585 598 10.1038/nrc2189 17646864

[B36] Podar K Chauhan D Anderson KC Bone marrow microenvironment and the identification of new targets for myeloma therapy Leukemia 2009 23 10 24 10.1038/leu.2008.259 18843284 PMC3418600

[B37] Edwards A Li J Atadja P Bhalla K Haura EB Effect of the histone deacetylase inhibitor LBH589 against epidermal growth factor receptor-dependent human lung cancer cells Mol Cancer Ther 2007 6 2515 2524 10.1158/1535-7163.MCT-06-0761 17876048

[B38] Lin TY Fenger J Murahari S Bear MD Kulp SK Wang D Chen CS Kisseberth WC London CA AR-42, a novel HDAC inhibitor, exhibits biologic activity against malignant mast cell lines via down-regulation of constitutively activated Kit Blood 2010 115 4217 4225 10.1182/blood-2009-07-231985 20233974 PMC3398750

[B39] Mitsiades CS Mitsiades N Munshi NC Anderson KC Focus on multiple myeloma Cancer Cell 2004 6 439 444 10.1016/j.ccr.2004.10.020 15542427

[B40] Sprynski AC Hose D Caillot L Reme T Shaughnessy JD Jr Barlogie B Seckinger A Moreaux J Hundemer M Jourdan M Meissner T Jauch A Mahtouk K Kassambara A Bertsch U Rossi JF Goldschmidt H Klein B The role of IGF-1 as a major growth factor for myeloma cell lines and the prognostic relevance of the expression of its receptor Blood 2009 113 4614 4626 10.1182/blood-2008-07-170464 19228610 PMC2691749

[B41] Mitsiades CS Mitsiades NS McMullan CJ Poulaki V Shringarpure R Akiyama M Hideshima T Chauhan D Joseph M Libermann TA García-Echeverría C Pearson MA Hofmann F Anderson KC Kung AL Inhibition of the insulin-like growth factor receptor-1 tyrosine kinase activity as a therapeutic strategy for multiple myeloma, other hematologic malignancies, and solid tumors Cancer Cell 2004 5 221 230 10.1016/S1535-6108(04)00050-9 15050914

[B42] Menu E van Valckenborgh E van Camp B Vanderkerken K The role of the insulin-like growth factor 1 receptor axis in multiple myeloma Arch Physiol Biochem 2009 115 49 57 10.1080/13813450902736583 19234898

[B43] Knuefermann C Lu Y Liu B Jin W Liang K Wu L Schmidt M Mills GB Mendelsohn J Fan Z HER2/PI-3K/Akt activation leads to a multidrug resistance in human breast adenocarcinoma cells Oncogene 2003 22 3205 3212 10.1038/sj.onc.1206394 12761490

[B44] Wang S Huang X Lee CK Liu B Elevated expression of erbB3 confers paclitaxel resistance in erbB2-overexpressing breast cancer cells via upregulation of Survivin Oncogene 2010 29 4225 4236 10.1038/onc.2010.180 20498641

